# Adapting Generative Large Language Models for Information Extraction from Unstructured Electronic Health Records in Residential Aged Care: A Comparative Analysis of Training Approaches

**DOI:** 10.1007/s41666-025-00190-z

**Published:** 2025-02-20

**Authors:** Dinithi Vithanage, Chao Deng, Lei Wang, Mengyang Yin, Mohammad Alkhalaf, Zhenyu Zhang, Yunshu Zhu, Ping Yu

**Affiliations:** 1 School of Computing and Information Technology, University of Wollongong, Wollongong, Australia; 2 School of Medical, Indigenous and Health Sciences, University of Wollongong, Wollongong, Australia; 3 Opal Healthcare, Sydney, Australia; 4 School of Computer Science, Qassim University, Qassim, Saudi Arabia

**Keywords:** Natural language processing, Generative large language models, Electronic health records, Information extraction, Llama

## Abstract

Information extraction (IE) of unstructured electronic health records is challenging due to the semantic complexity of textual data. Generative large language models (LLMs) offer promising solutions to address this challenge. However, identifying the best training methods to adapt LLMs for IE in residential aged care settings remains underexplored. This research addresses this challenge by evaluating the effects of zero-shot and few-shot learning, both with and without parameter-efficient fine-tuning (PEFT) and retrieval-augmented generation (RAG) using Llama 3.1-8B. The study performed named entity recognition (NER) to nursing notes from Australian aged care facilities (RACFs), focusing on agitation in dementia and malnutrition risk factors. Performance evaluation includes accuracy, macro-averaged precision, recall, and F1 score. We used non-parametric statistical methods to compare if the differences were statistically significant. Results show that zero-shot and few-shot learning, whether combined with PEFT or RAG, achieve comparable performance across the clinical domains when the same prompting template is used. Few-shot learning significantly outperforms zero-shot learning when neither PEFT nor RAG is applied. Notably, PEFT significantly improves model performance in both zero-shot and few-shot learning; however, RAG significantly improves performance only in few-shot learning. After PEFT, the performance of zero-shot learning reaches a comparable level with few-shot learning. However, few-shot learning with RAG significantly outperforms zero-shot learning with RAG. We also found a similar level of performance between few-shot learning with RAG and zero-shot learning with PEFT. These findings provide valuable insights for researchers, practitioners, and stakeholders to optimize the use of generative LLMs in clinical IE.

**Supplementary Information:**

The online version contains supplementary material available at 10.1007/s41666-025-00190-z.

## Introduction

The rapid digitization of healthcare has led to the widespread adoption of electronic health records (EHRs), which store vast amounts of patient data. Electronic health records include structured data, such as demographic information and laboratory results, and unstructured data, such as clinical notes. Structured data, characterized by its predefined format, can be easily queried and utilized. In contrast, unstructured data, often found in free-text formats, encompass a significant portion of comprehensive and rich patient data [1]. These narratives, written by healthcare professionals, provide detailed insights into patient conditions, diagnoses, treatments, and outcomes. However, the large volume and complex nature of unstructured EHRs have posed significant challenges in effectively utilizing this valuable information [2]. Information extraction (IE) offers a promising solution to address these challenges [3].

Information extraction (IE) involves transforming unstructured text into structured data, such as entities (e.g., patient names and symptoms), relationships (e.g., between medications and diagnoses), or events (e.g., clinical procedures). This structured data enables more effective analysis of the rich information embedded in unstructured text [4]. However, developing IE tools for these transformation processes poses considerable challenges due to the variability in clinical terminologies, the context-dependent nature of meanings, and the sheer volume of unstructured text [5].

To address these complexities and fully realize the potential of unstructured data in healthcare, adaptable and scalable automated IE tools are required. Natural language processing (NLP) techniques, such as named entity recognition (NER), play a key role in automating these processes, offering solutions to these challenges [1]. Named entity recognition identifies and classifies entities within text, such as symptoms, diagnoses, and treatments [1, 6, 7]. Previous research in clinical NLP has largely focused on clinical IE, with BERT-based models demonstrating state-of-the-art performance in NER tasks [8–12]. These models have proven highly effective in processing and analyzing complex clinical text. Despite this progress, the diverse and complex ways in which healthcare professionals use natural language pose significant challenges to achieving effective clinical IE [1]. Addressing these challenges requires advanced approaches for clinical IE that can adapt to the linguistic diversity in clinical narratives while maintaining high accuracy and efficiency.

The recent advancements in generative large language models (LLMs), such as GPT variants, T5, OPT, and Llama [13–15], have showcased their ability to generate human-like text and even surpass human-level performance in certain NLP tasks: text summarization, question answering, and machine translation [1, 16, 17]. However, the application of generative LLMs to clinical IE remains in its early stages, with significant gaps. These include the limited comparison of training methods using actual clinical data to identify optimal training approaches for adapting these models, particularly for mining free-text nursing progress notes, which has sparked debates within the research community. To address this knowledge gap, it is essential to explore and refine training methods that effectively adapt generative LLMs for optimal performance in clinical IE for nursing care in various settings, including residential aged care (RAC).

## Related Work

Existing studies adapt generative LLMs to clinical IE tasks through various training methods. Common training methods are prompt engineering with zero-shot and few-shot learning, parameter-efficient fine-tuning (PEFT), and retrieval-augmented generation (RAG).

### Prompt Engineering

A prompt is an input that a user enters to instruct an LLM to automatically generate sequential output [18]. An LLM uses pattern matching to identify the relationships between the words, phrases, and concepts in the prompt and connect these with the patterns learned from the previous training. It then uses natural language generation to respond in a human-understandable format. Prompts enable the model to adapt and comprehend specific information in a new domain, leveraging its learned knowledge stored within the pre-trained models like Llama and GPT, thereby expanding the model’s applicability and effectiveness [19]. Prompt learning reduces the need to introduce new parameters or extensive retraining of the model using labeled data for various tasks, thus improving efficiency and reducing computational resources required for training for LLMs. Common prompt training methods include zero-shot and few-shot learning.

#### Zero-Shot Learning

Zero-shot learning uses single-prompt instruction to train LLMs for specific NLP tasks, directly applying previously trained models to predict both seen and unseen classes without using any labeled training instances [20]. Zero-shot learning with generative LLMs has achieved impressive performance in NLP tasks including clinical IE [19–23], which proves the feasibility of using LLMs to extract structured information from clinical data with minimal technical expertise.

#### Few-Shot Learning

Few-shot learning, also coined as in-context learning, refers to the ability of LLMs to perform tasks guided by a small set of representative examples provided in the prompt [24, 25]. These in-context examples not only teach the LLM the mapping from inputs to outputs but also activate the LLM’s parametric knowledge. Only requiring a handful of labeled training examples is a clear advantage of few-shot learning, making it data-efficient and accessible to knowledge domain users without expertise in machine learning [24]. Few-shot learning is particularly useful in situations where annotating text data is not convenient or expensive. Domain experts can quickly create a generative AI system for a new task by only providing a few examples. Importantly, few-shot learning does not change the underlying model weights [26]. This allows for efficient adaptation to new tasks without risking the loss of previously learned knowledge. However, the performance of few-shot learning varies and is highly task-dependent [24]. Its accuracy is also sensitive to prompt templates and in-context examples. Prior research finds that using semantically similar in-context examples to those with prior success can significantly enhance the performance of few-shot learning, especially in clinical IE [19, 27].

### Parameter-Efficient Fine-tuning

Parameter-efficient fine-tuning involves modifying the LLM, or the parameters used to train the LLM, to improve model response to the same prompt [18]. Fine-tuning changes a model’s weight, thus, the model’s behavior to perform better at a specific task [28–31]. Full fine-tuning will fine-tune all layers of the pre-trained model, which can be computationally expensive and may lead to catastrophic forgetting, i.e., the model forgets the knowledge it gained during pre-training. Thus, it may significantly increase the cost of computational resources and computational skill sets. Parameter-efficient fine-tuning only fine-tunes a small number of (extra) parameters while freezing most parameters of the pre-trained LLMs. It thus overcomes the computational resource constraint and catastrophic forgetting problem observed in the full-scope fine-tuning of LLM.

Low-Rank Adaptation (LoRA) is a PEFT technique designed to improve training efficiency for LLMs [29, 31, 32]. It freezes the weight of per-trained LLMs and inserts low-rank decomposition matrices into the transformer layers. Previous research has demonstrated that LoRA can allow the fine-tuning process to focus on crucial parameters specific to the target task or domain (e.g., clinical IE), thus optimizing the model’s performance without extensive resource requirements or overfitting concerns [33, 34]. By focusing on PEFT, LoRA minimizes the dependency on extensive labeled data for model optimization, maximizing the utility of available data and making the fine-tuning process more effective and feasible in scenarios with limited annotated datasets.

### Retrieval-Augmented Generation

RAG enhances LLMs by enabling access to real-time, relevant information from external knowledge sources, improving response accuracy and relevance [35–38]. The process includes indexing, where data are converted into embeddings for efficient retrieval; retrieval, where the system identifies the most pertinent documents based on a user query; augmentation, where the retrieved information enriches the query’s context; and generation, where the LLMs produce a response informed by both the original query and the augmented data. Previous research in NLP tasks, especially clinical IE, has demonstrated that this dynamic approach addresses traditional LLM limitations, ensuring up-to-date and contextually accurate responses [35, 39].

Despite the promising outcomes of existing LLMs, they exhibit critical limitations in performing clinical IE. These limitations underscore a significant knowledge gap that must be addressed for the practical deployment of LLMs to assist clinicians, particularly in mining free-text nursing progress notes within the Australian RAC context: (1) a lack of comprehensive comparisons, as current studies focus primarily on individual training methods, offering limited insights into their relative effectiveness for clinical IE across different contexts; and (2) limited applicability to real-world clinical settings, as most studies rely on synthetic datasets that fail to capture the complexities and challenges inherent in real-world clinical documents, particularly free-text nursing documents.

To address this critical gap, we conducted a study to systematically compare the performance of various LLM training methods for clinical IE. These methods include prompt engineering with zero-shot and few-shot learning, both with and without PEFT and RAG. Our investigation utilized nursing progress notes collected from residential aged care facilities (RACFs) in Australia. This dataset is particularly valuable for two reasons: (1) its depth and richness, offering detailed, narrative accounts of daily care that encompass critical information about residents’ physical, emotional, and social well-being. This richness enables the extraction of diverse and valuable clinical insight [40, 41], making it a valuable resource for IE research; and (2) its real-world relevance. Unlike synthetic datasets, this dataset represents the inherent complexity and variability of real-world clinical environments, offering a robust resource for evaluating the effectiveness of training methods in adapting LLMs for use in nursing care in RACFs.

To the best of our knowledge, no prior study has systematically compared training methods for generative LLMs in IE using real-world data from RAC settings. This study addresses an under-studied clinical setting in literature, providing new insights into the potential of generative LLMs to meet the demands of aged care.

We selected NER as the primary IE task, focusing on two clinical domains: agitation in dementia and malnutrition risk factors. These domains were chosen for their clinical significance and the nature of the data recorded in free-text nursing progress notes. For this reason, the research team curated labeled datasets to enable model training, validation, and testing [42–44]. The clinical domains encompassed a diverse range of clinical entities, with entity counts ranging from 37 to 83 (see Table 1).

**Table 1:** Clinical domains and entities for NER

Clinical domains	Clinical entities	Number of clinical entities
Agitation in dementia	Disruptive vocalization, verbally aggressive behavior, arguing, complaining, cursing, threat, using abusive language, using accusatory language, using foul language, using hostile language, using obscene language, using profane language, verbally nonaggressive behavior, ceaseless talking, constant repetition of word, constant unwarranted requests for attention, constant unwarranted requests for help, constant unwarranted requests for reassurance, echolalia, groaning, grunting, howling, making bizarre noise, rambling, repetitive questioning, roaring, screaming, shouting, speaking in excessively loud voice, emotional distress, anger, frustration, irritability, mood swing, negativism, outburst, physically aggressive behavior, biting, destroying property, fighting, grabbing, hitting, hurting self, hurting someone, kicking, pushing, resisting, scratching, shoving, slamming, spitting on people, staring, striking people, tearing, throwing object, physically nonaggressive behavior, constant manipulation of object, fidgeting, gesturing, hand wringing, inappropriate dressing, inappropriate handling object, inappropriate undressing, pacing, pointing finger, repetitive physical mannerism, restlessness, rocking, rummaging, searching, wandering, bruxism, resisting, punching, absconding, calling out, physical agitation, facial grimacing, moving furniture, hoard items, intrusive of others privacy, gets up and down from constantly, urinating on the floor	83
Risk factors for malnutrition	Anxiety, bowel blockage, cancer, chest infection, chronic wound, confusion, constipation, delirium, dementia, depression, diabetes, diarrhea, difficulty swallowing, dysphagia, eating disorder, food preference, frailty, gastritis, heart disease, HIV, hospital admission, isolation, kidney disease, liver disease, malabsorption medication, nausea, Parkinson, pneumonia, poor appetite, poor intake, poor oral health, pressure ulcer, sepsis, stroke, suboptimal intake, surgery, vomiting	37

We designed experiments to test the research hypotheses outlined in Table 2, guiding our evaluation of training methods for adapting LLMs to clinical IE.

**Table 2:** Research hypotheses in the study

Hypothesis	Null hypothesis	Alternative hypothesis
1	Zero-shot and few-shot learning with similar prompting templates have different levels of performance when applied to NER for various clinical domains	Zero-shot and few-shot learning with similar prompting templates have the same level of performance when applied to NER for various clinical domains
2	Few-shot learning does not perform better than zero-shot learning for the same NER task	Few-shot learning performs better than zero-shot learning for the same clinical NER task
3	Parameter-efficient fine-tuning does not improve the performance of either zero-shot or few-shot learning	Parameter-efficient fine-tuning improves the performance of both zero-shot and few-shot learning
4	Zero-shot learning does not reach the same level of performance as few-shot learning for the same NER task after PEFT	Zero-shot learning reaches the same level of performance as few-shot learning for the same NER task after PEFT
5	Fine-tuning for one clinical domain does not impact model performance across other clinical domains in NER	Fine-tuning for one clinical domain impacts model performance across other clinical domains in NER
6	RAG does not improve the performance of both zero-shot and few-shot learning	RAG improves the performance of both zero-shot and few-shot learning
7	Zero-shot learning does not reach the same level of performance as few-shot learning for the same NER task with RAG	Zero-shot learning reaches the same level of performance as few-shot learning for the same NER task with RAG
8	Few-shot learning with RAG and zero-shot learning with PEFT do not have the same performance levels in NER	Few-shot learning with RAG and zero-shot learning with PEFT have the same performance levels in NER

## Methodology

We conducted the experiment in seven stages: generative LLM selection, data set selection, data preprocessing, designing prompt templates for zero-shot and few-short learning, training methods execution, model performance evaluation, and statistical analysis.

### Ethics Approval

The Human Research Ethics Committee of the University of Wollongong approved the study (Ethics Number 2019/159).

### Generative LLM Selection

We selected the Llama 3.1-8B-parameter model as the generative LLM. The selection considered the following factors: (1) the optimal model in terms of open source and favorable review at the time of the experiment; (2) practical considerations regarding the availability of GPU resources; (3) feasibility for local server deployment, convenience, and control over usage; (4) compliance with health data privacy regulations in Australia; (5) the presence of diverse variants spawned through fine-tuning, including Alpaca, Baizem, Koala, and Vicuna [15]. We obtained the Llama 3.1 8B-parameter model from the Hugging Face repository (https://huggingface.co/meta-llama/Meta-Llama-3.1-8B-Instruct).

### Data Set Selection

De-identified demographic data and free-text nursing progress notes were collected for the same population of older people living in 40 RACFs in New South Wales, Australia, from 2019 to 2021. The structured demographic information included masked sequence numbers for client de-identification, age, and gender. The unstructured nursing notes included nursing assessment and progress reporting. They documented clients’ daily activities, care staff’s clinical observations, assessments of client’s care needs (including risk factors), and carer interventions.

### Data Preprocessing

Text preprocessing involved the removal of URLs and non-textual characters, such as extra delimiters and empty spaces in the dataset. We made a choice not to exclude stop words because many of them, like “a,” “be,” “very,” and “should,” held semantic relevance to the content [45].

### Designing Prompt Templates for Zero-Shot and Few-Short Learning in Each Clinical Task

**Fig. 1:**
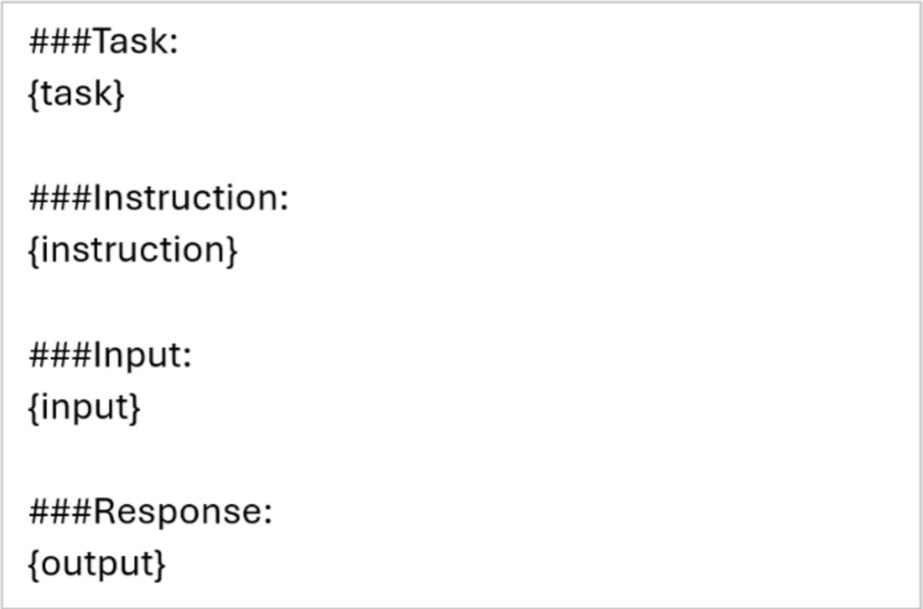
Prompt template inspired by Abdallaha et al. [46]

In this study first, we selected prompt-based training via zero-shot and few-shot learning and then the few-shot learning. We adopted the zero-shot and few-shot learning templates inspired by Abdallaha et al. [46] to construct our prompt (see Fig. 1).

The final prompts used in our experiment are listed in Table 3. The example results generated from the final prompts are showcased in Supplementary Table 1.

**Table 3:** Prompts used in this study

Prompt learning technique	Clinical domains	Prompt
Zero-shot	Agitation in dementia	**###Task:** Review nursing progress notes for agitation symptoms in dementia As a nursing expert, you are required to review a nursing progress note for a resident with dementia in a residential aged care facility. Your review should focus on identifying symptoms of agitation associated with dementia as documented in the note Symptoms of agitation in dementia: Disruptive vocalisation, verbally aggressive behaviour, arguing, complaining, cursing, threat, using abusive language, using accusatory language, using foul language, using hostile language, using obscene language, using profane language, verbally nonaggressive behaviour, ceaseless talking, constant repetition of word, constant unwarranted requests for attention, constant unwarranted requests for help, constant unwarranted requests for reassurance, echolalia, groaning, grunting, howling, making bizarre noise, rambling, repetitive questioning, roaring, screaming, shouting, speaking in excessively loud voice, emotional distress, anger, frustration, irritability, mood swing, negativism, outburst, physically aggressive behaviour, biting, destroying property, fighting, grabbing, hitting, hurting self, hurting someone, kicking, pushing, resisting, scratching, shoving, slamming, spitting on people, staring, striking people, tearing, throwing object, physically nonaggressive behaviour, constant manipulation of object, fidgeting, gesturing, hand wringing, inappropriate dressing, inappropriate handling object, inappropriate undressing, pacing, pointing finger, repetitive physical mannerism, restlessness, rocking, rummaging, searching, wandering, bruxism, resisting, punching, absconding, calling out, physical agitation, facial grimacing, moving furniture, hoard items, intrusive of others privacy, gets up and down from constantly, urinating on the floor **###Instrcuctions:** 1. Identify symptoms: Examine the progress note to identify any symptoms of agitation, including those listed above. If symptoms that are not explicitly listed are observed, include them as well, ensuring they are relevant to agitation in dementia 2. List identified symptoms: If agitation symptoms are evident, provide a complete and concise list of all identified symptoms **### Input: < **input text** > ** **### Response: < **output text** > **
Few-shot	Agitation in dementia	**###Task:** Review nursing progress notes for agitation symptoms in dementia As a nursing expert, you are required to review a nursing progress note for a resident with dementia in a residential aged care facility. Your review should focus on identifying symptoms of agitation associated with dementia as documented in the note Symptoms of agitation in dementia: Disruptive vocalisation, verbally aggressive behaviour, arguing, complaining, cursing, threat, using abusive language, using accusatory language, using foul language, using hostile language, using obscene language, using profane language, verbally nonaggressive behaviour, ceaseless talking, constant repetition of word, constant unwarranted requests for attention, constant unwarranted requests for help, constant unwarranted requests for reassurance, echolalia, groaning, grunting, howling, making bizarre noise, rambling, repetitive questioning, roaring, screaming, shouting, speaking in excessively loud voice, emotional distress, anger, frustration, irritability, mood swing, negativism, outburst, physically aggressive behaviour, biting, destroying property, fighting, grabbing, hitting, hurting self, hurting someone, kicking, pushing, resisting, scratching, shoving, slamming, spitting on people, staring, striking people, tearing, throwing object, physically nonaggressive behaviour, constant manipulation of object, fidgeting, gesturing, hand wringing, inappropriate dressing, inappropriate handling object, inappropriate undressing, pacing, pointing finger, repetitive physical mannerism, restlessness, rocking, rummaging, searching, wandering, bruxism, resisting, punching, absconding, calling out, physical agitation, facial grimacing, moving furniture, hoard items, intrusive of others privacy, gets up and down from constantly, urinating on the floor **###Instrcuctions:** 1. Identify symptoms: Examine the progress note to identify any symptoms of agitation, including those listed above. If symptoms that are not explicitly listed are observed, include them as well, ensuring they are relevant to agitation in dementia 2. List identified symptoms: If agitation symptoms are evident, provide a complete and concise list of all identified symptoms Example cases: Example 1: Identified symptoms: physical agitation/aggression, verbal disruption, calling out, screaming Progress note: "Resident with vascular dementia exhibits confusion and disorientation alongside significant physical agitation/aggression and verbal behaviors, including verbal disruption, calling out, and screaming. These behaviors disturb others. Staff provide reassurance and distraction strategies such as music therapy and card games. During heightened distress, her daughter is contacted for support." Example 2: Identified symptoms: wandering, frightening others, arguing, refusing care Progress note: "Resident frequently wanders into others' rooms, mistaking them for her own. This behavior frightens others and results in arguments. The resident refuses care for hygiene and continence management, requiring staff intervention and redirection. Mealtimes involve guided supervision to ensure safety." Example 3: Identified symptoms: punching Progress note: "Resident's advancing dementia has led to increased physical aggression, particularly punching during interactions with staff when she feels threatened or confused. Staff are implementing strategies to provide reassurance and maintain a safe environment." Please use this structured approach to complete your review of the nursing progress note **### Input: < **input text** > ** **### Response: < **output text** > **
Zero-shot	Malnutrition risk factors	**###Task:** Review nursing progress notes for malnutrition risk factors in dementia As a nursing expert, you are required to review a nursing progress note for a resident in a residential aged care facility. Your review should focus on identifying potential risk factors for malnutrition as documented in the note Potential malnutrition risk factors: Anxiety, bowel blockage, cancer, chest infection, chronic wound, confusion, constipation, delirium, dementia, depression, diabetes, diarrhea, difficulty swallowing, dysphagia, eating disorder, food preference, frailty, gastritis, heart disease, HIV, hospital admission, isolation, kidney disease, liver disease, malabsorption medication, nausea, Parkinson, pneumonia, poor appetite, poor intake, poor oral health, pressure ulcer, sepsis, stroke, suboptimal intake, surgery, vomiting **###Instructions:** 1. Identify risk factors: Examine the progress note to identify any malnutrition risk factors, including those listed above. If risk factors not explicitly listed are observed, include them as well, ensuring they are relevant to malnutrition 2. List identified risk factors: If any malnutrition risk factors are evident, provide a complete and concise list of the identified risk factors **### Input: < **input text** > ** **### Response: < **output text** > **
Few-shot	Malnutrition risk factors	**###Task:** Review nursing progress notes for malnutrition risk factors in dementia As a nursing expert, you are required to review a nursing progress note for a resident in a residential aged care facility. Your review should focus on identifying potential risk factors for malnutrition as documented in the note Potential malnutrition risk factors: Anxiety, bowel blockage, cancer, chest infection, chronic wound, confusion, constipation, delirium, dementia, depression, diabetes, diarrhea, difficulty swallowing, dysphagia, eating disorder, food preference, frailty, gastritis, heart disease, HIV, hospital admission, isolation, kidney disease, liver disease, malabsorption medication, nausea, Parkinson, pneumonia, poor appetite, poor intake, poor oral health, pressure ulcer, sepsis, stroke, suboptimal intake, surgery, vomiting **###Instructions:** 1. Identify risk factors: Examine the progress note to identify any malnutrition risk factors, including those listed above. If risk factors not explicitly listed are observed, include them as well, ensuring they are relevant to malnutrition 2. List identified risk factors: If any malnutrition risk factors are evident, provide a complete and concise list of the identified risk factors Example Cases: Example 1: Identified risk factor: confusion Progress Note: “Resident requires comprehensive assistance with his hygiene and toileting needs due to his confusion. He experiences incontinence of both urine and feces, necessitating the use of pads around the clock. A nurse is responsible for assisting him with toileting, changing his pads, cleansing his groin area, and applying barrier cream to mitigate the risk of skin issues or breakdown.” Example 2: Identified risk factor: constipation Progress note: “Resident requires the assistance of a nurse for his hygiene and toileting needs, primarily due to his unsteady gait. He utilizes pads due to incontinence issues. Additionally, he is prone to constipation and receives aperients as necessary. The current intervention measures in place have proven to be effective.” Please use this structured approach to complete your review of the nursing progress note **### Input: < **input text** > ** **### Response: < **output text** > **

### Training Methods Execution

We selected PEFT, prompt-based learning (without PEFT or RAG), and RAG to test the LLM’s (Llama 3.1) ability to adapt, generalize, and optimize performance in clinical IE tasks.

#### Experiment Setup

##### Parameter Efficient Fine-tuning with LoRA on Llama 3.1

We used the PEFT method to fine-tune the Llama model. The experiment was conducted on four NVIDIA RTX-A5000, each equipped with 24 GB of memory. The use of multiple GPUs not only accelerated the training process but also ensured that the model could be fine-tuned within a reasonable timeframe. Our software environment was Ubuntu 18.04, the programming language was Python 3.10.0, and the deep learning framework was Pytorch 2.0.0. Instruction data points were employed during the PEFT process, and the hyperparameter settings are listed in Table 4. In these hyperparameter settings, batch size refers to the number of training examples processed in a single iteration of the model’s training process. However, due to the memory limitations of the available hardware, we employed a micro-batch size strategy, which splits the full batch into smaller chunks and processes them sequentially. This allowed us to handle the memory load efficiently while still achieving the target batch size of 128 with the Llama model [47, 48]. To further optimize memory usage, we applied 8-bit quantization using the BitsandBytes library, compressing the model weights into a lower precision format that fits within our GPU memory [35]. This same quantization strategy was utilized for other experiments as well, including (1) prompt-based learning without PEFT or RAG and (2) RAG, which are discussed in subsequent sections. The combination of micro-batch size and quantization was essential for effectively training the model within the hardware constraints, ensuring the smooth and efficient execution of our experiments.

**Table 4:** Hyperparameters used in PEFT

Settings	Parameters
Batch size	128
Micro batch size	4
LoRA rank	8
LoRA alpaca	16
LoRA dropout	0.05
Learning rate	3e-4
Training steps	300
Optimizer	AdamW
Trainable parameters (%)	< 0.01%

Llama 3.1 model’s maximum token limit is 8192, which was large enough to encompass the available tokens for each nursing note. During the fine-tuning process, the model iteratively processed each note within the defined token limit. The annotated datasets were used from the previous studies. The dataset for agitation in dementia was derived from the studies by Zhu et al. [42, 43], and the dataset for malnutrition was based on the work of Alkhalaf et al. [44]. We randomly divided this labeled data (ensuring no overlapping free-text notes within the labeled dataset) detailed in Table 5 into 80% training, 10% validation, and 10% testing sets for each clinical task. This process was repeated three times to mitigate potential bias from different data splits, and cross-validation was conducted to achieve reliable results [49]. The dedicated test datasets were explicitly used to assess the other training methods including prompt-based learning (without PEFT or RAG) and RAG, allowing for a comprehensive comparison and analysis of the NER task to test the research hypotheses outlined in Table 1.

**Table 5:** Number of labeled data and file size for each clinical task

Clinical domains	Training + validation data	File size	Output model
Agitation in dementia	3000 nursing notes	5.89 MB	Agitation in dementia with specialized PEFT of Lama 3.1
Malnutrition risk factors	2850 nursing notes	972 KB	Malnutrition risk factors with specialized PEFT of Lama 3.1

We used the prompts delineated in Table 3 to evaluate the test data. First, we conducted zero-shot learning with PEFT on Llama 3.1 across the test datasets for NER. This was followed by few-shot learning with PEFT on Llama 3.1 across the same test datasets for NER. Furthermore, to address variability in results caused by model randomness, multiple evaluation sessions were conducted over three weeks, with results being collected at three distinct time points. We applied the same approach of repeated evaluation sessions for consistency in two other experiments—prompt training without PEFT or RAG and RAG—allowing for a more reliable and comparable evaluation across all methods.

##### Prompt-Based Learning with Zero-Shot and Few-Shot Learning on Llama 3.1

The experiment was conducted in an environment similar to the one described in the “Parameter Efficient Fine-tuning with LoRA on Llama 3.1” section, with the main difference being the number of GPUs used. Since prompt-based learning with the original Llama 3.1 requires fewer computational resources due to the absence of fine-tuning [50], we were able to utilize a single GPU with 24 GB. The same process was employed for the experiment with RAG, as no fine-tuning was involved in the RAG process. We employed Llama 3.1, utilizing zero-shot and few-shot learning prompts as outlined in Table 3. To prevent model contamination, we approached the NER task in two distinct steps. Initially, we employed the Llama 3.1 model that we directly downloaded from the Hugging Face repository, for zero-shot learning. Afterward, we downloaded a new copy of the same model from the same repository for few-shot learning. The Llama 3.1 model has a maximum token limit of 8192, and none of the test notes (see the sections titled “Parameter Efficient Fine-tuning with LoRA on Llama 3.1” for details about the test datasets) exceeded this token count during evaluation. Consequently, the model processed each note iteratively within this defined token limit during testing.

##### ***RAG with Llama 3.1***

The experiment for Llama 3.1, with RAG, began with data preparation, utilizing a JSON file containing nursing notes that encompass all the entities (refer to Table 1). The clinical domains, and the number of nursing notes provided in the JSON file, are detailed in Table 6.

**Table 6:** Data used in the RAG

Clinical domains	Number of nursing notes	File size
Agitation in dementia	83	14 KB
Malnutrition risk factors	37	10 KB

This data was input into an embedding model. The embedding model (https://huggingface.co/sentence-transformers/all-mpnet-base-v2) generated embeddings from the data, which were then stored in a vector store. A query was formulated by combining the prompt with zero-shot or few-shot examples. The query was applied to the dedicated test datasets (see the sections titled “Parameter Efficient Fine-tuning with LoRA on Llama 3.1” for details about the test datasets). The retriever used the query to fetch relevant data from the vector store. The retrieval process, managed by LlamaIndex, determined relevance based on the similarity between the query and the stored embeddings, which were then used by the LLM to generate the output [36, 51].

### Model Performance Evaluation

To assess model performance on the clinical NER tasks, we utilized micro-average precision, recall, F1 score, and accuracy, which were more appropriate for imbalanced class distributions [52, 53]. We set the threshold value as 0.5 for the label alignment in the evaluation process, following prior works [49, 50]. We calculated the BERTscore to obtain precision, recall, and F1 score following the methods by Li et al. [54]. We then used the Scikit-learn library, which offers built-in metrics for IE, to calculate the micro-average F1 score, precision, and recall [55, 56]. The model outputs, generated in JSON format, were compared against the annotated ground truth data, which were also stored in JSON format. An extracted entity or phrase was considered correct if it overlapped with the text and conveyed the exact or highly similar meaning of the annotated ground truth entity or phrase. For instance, if the ground truth annotation is “shouting” as a symptom of agitation in dementia, and the model output is also “shouting,” it is evaluated as an exact match. Alternatively, if the ground truth annotation is “reject meals” and the model output is “refuse meals,” it is considered a match based on semantic similarity, as the two phrases share the same meaning.

### Statistical Analysis

As the measurement indicators, including accuracy, precision, recall, and F1 score, were not normally distributed, we utilized the non-parametric Kruskal–Wallis test for comparing results across three or more independent groups and the Mann–Whitney U test for comparing two independent groups to test the hypotheses, as suggested by the previous research [46, 57]. A significant difference is decided if the *p*-value is smaller than 0.05. The statistical analysis results are presented in Supplementary Table 2.

## Results

In this section, we present the study’s findings. The experiments were conducted three times for cross-validation, and the results showed no significant differences in performance across the three runs (Supplementary Table 3, *p* > 0.05).

### Results of Testing Hypothesis 1: Zero-Short and Few-Shot Learning with Similar Prompting Templates May (or May Not) Exhibit the Same Level of Performance when Applied to NER for Various Clinical Domains

To evaluate Hypothesis 1, we compared (1) the performance of zero-shot learning (without PEFT or RAG), (2) the performance of zero-shot learning with PEFT, (3) the performance of zero-shot learning with RAG, (4) the performance of few-shot learning (without PEFT or RAG), (5) the performance of few-shot learning with PEFT, and (6) the performance of few-shot learning with RAG across the agitation in dementia and malnutrition risk factors in NER.

#### Comparing the Performance of Zero-Shot Learning (Without PEFT or RAG) for Agitation in Dementia and Malnutrition Risk Factors in NER

**Fig. 2:**
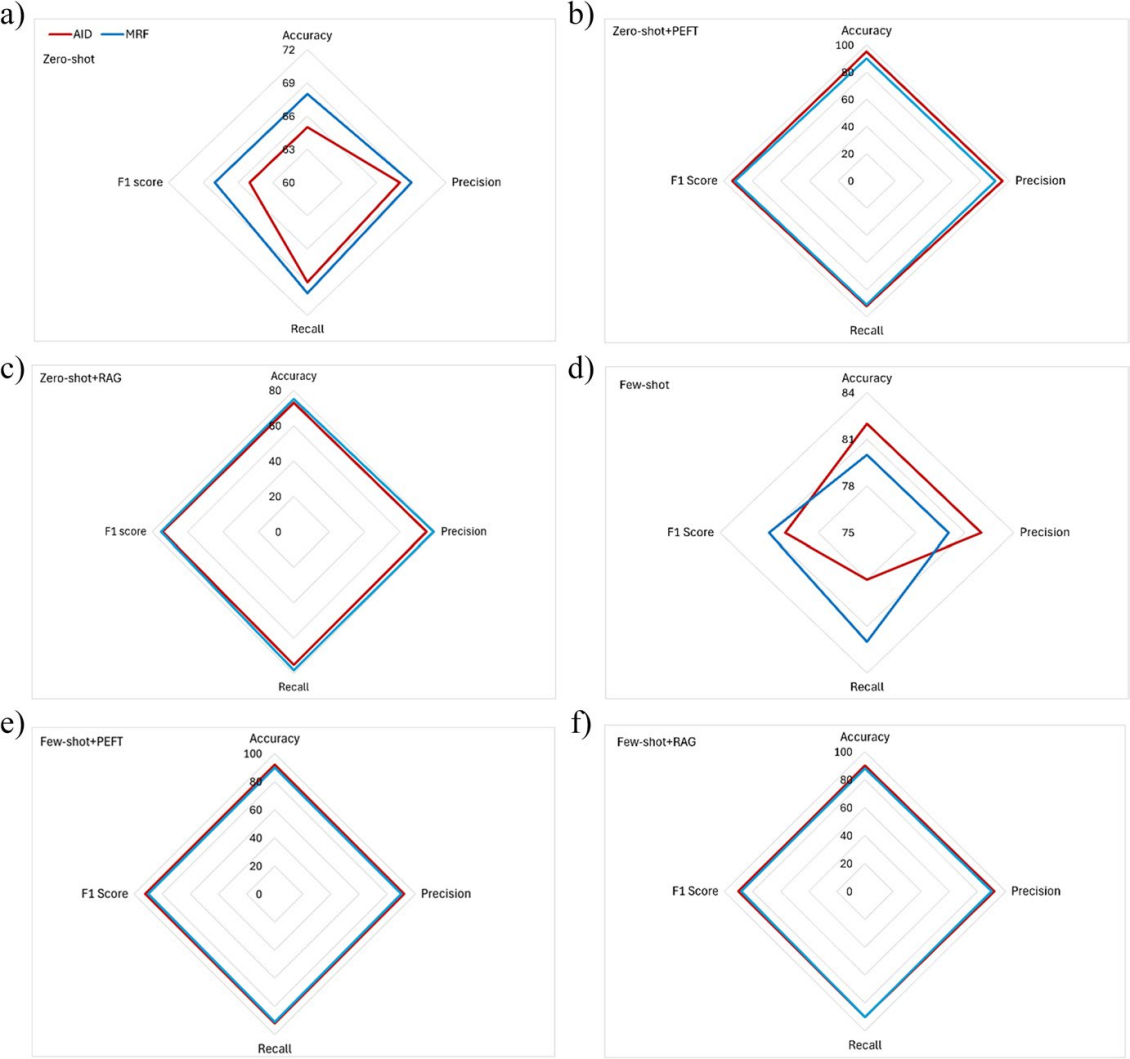
Comparative evaluation of model performance for agitation in dementia and malnutrition risk factors in NER with the following training methods: **a** zero-short learning, **b** zero-short learning with PEFT, **c** zero-short learning with RAG, **d** few-short learning, **e** few-short learning with PEFT, and **f** few-short learning with RAG. Note: “AID” denotes agitation in dementia, and “MRF” denotes malnutrition risk factors. The same notation applies to the figures that follow. Note: “ + PEFT” denotes with PEFT. The same notation applies to the figures that follow. Note: “ + RAG” denotes with RAG. The same notation applies to the figures that follow

There is no statistically significant difference in accuracy, precision, recall, and F1 score between the agitation in dementia and malnutrition risk factors undertaking this training method (Fig. 2a, p > 0.05).

#### Comparing the Performance of Zero-Shot Learning with PEFT for Agitation in Dementia and Malnutrition Risk Factors in NER

Once again, no statistically significant difference is found in accuracy, precision, recall, and F1 score between the agitation in dementia and malnutrition risk factors undertaking this training method (Fig. 2b, p > 0.05).

#### Comparing the Performance of Zero-Shot Learning with RAG for Agitation in Dementia and Malnutrition Risk Factors in NER

No statistically significant difference is found in accuracy, precision, recall, and F1 score between the agitation in dementia and malnutrition risk factors undertaking this training method (Fig. 2c, p > 0.05).

#### Comparing the Performance of Few-Shot Learning (Without PEFT or RAG) for Agitation in Dementia and Malnutrition Risk Factors in NER

No statistically significant difference is found in accuracy, precision, recall, and F1 score between the agitation in dementia and malnutrition risk factors under this training method (Fig. 2d, p > 0.05).

#### Comparing the Performance of Few-Shot Learning with PEFT for the Agitation in Dementia and Malnutrition Risk Factors in NER

Again, no statistically significant difference is found in accuracy, precision, recall, and F1 score among the agitation in dementia and malnutrition risk factors (Fig. 2e, p > 0.05).

#### Comparing the Performance of Few-Shot Learning with RAG for Agitation in Dementia and Malnutrition Risk Factors in NER

No statistically significant difference is found in accuracy, precision, recall, and F1 score between the agitation in dementia and malnutrition risk factors undertaking this training method (Fig. 2f, p > 0.05).

### Results of Testing Hypothesis 2: Few-Shot Learning May (or May Not) Perform Better than Zero-Shot Learning for the Same NER Task

**Fig. 3:**
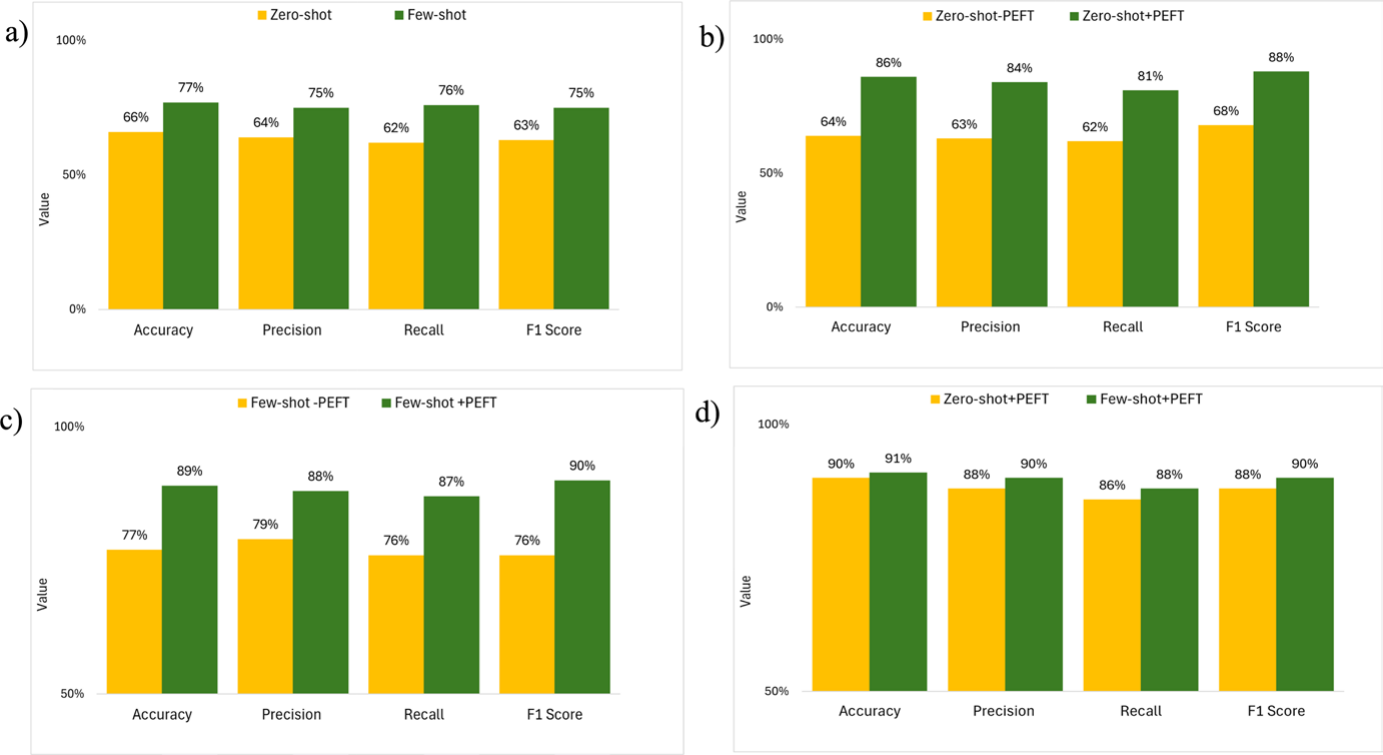
Comparative evaluation of model performance for NER with the following training methods: **a** zero-shot learning versus few-shot learning, **b** zero-shot learning versus zero-shot learning with PEFT, **c** few-shot learning versus few-shot learning with PEFT, and **d** zero-shot learning versus few-shot learning with PEFT. Note: “-PEFT” denotes without PEFT. The same notation applies to the figures that follow

To evaluate Hypothesis 2, we compared the performance of zero-shot and few-shot learning (without PEFT and RAG) for all entities in agitation in dementia and malnutrition risk factors in NER. Few-shot learning significantly improves model accuracy, precision, recall, and F1 score in NER than zero-shot learning (Fig. 3a, p < 0.05). The level of improvement includes a 17% increase in model accuracy, a 17% increase in precision, a 23% increase in recall, and a 19% increase in F1 score.

### Results of Testing Hypothesis 3: Parameter-Efficient Finetuning May (or May Not) Improve Both Zero-Shot and Few-Shot Learning Performance

To evaluate Hypothesis 3, we compared (1) the performance of the zero-shot learning model without PEFT and with PEFT and (2) the performance of the few-shot learning model without PEFT and with PEFT for all entities in agitation in dementia and malnutrition risk factors in NER.

#### Comparing the Performance of the Zero-Shot Learning Model Without PEFT and with PEFT for NER

Zero-shot learning with PEFT significantly improves model accuracy, precision, recall, and F1 score in NER (Fig. 3b, p < 0.05). The level of improvement is as follows: a 26% increase in model accuracy, a 33% increase in precision, a 31% increase in recall, and a 29% increase in F1 score.

#### Comparing the Performance of the Few-Shot Learning Model Without PEFT and with PEFT for NER

Few-shot learning with PEFT significantly improves model accuracy, precision, recall, and F1 score in NER (Fig. 3c, p < 0.05). The level of improvement is as follows: a 16% increase in model accuracy, an 11% increase in precision, a 14% increase in recall, and an 18% increase in F1 score.

### Results of Testing Hypothesis 4: Zero-Shot Learning May (or May Not) Reach the Same Level of Performance as Few-Shot Learning for the NER with PEFT

To evaluate Hypothesis 4, we compared the performance of zero-shot and few-shot learning with PEFT for NER. Although no statistically significant difference is found in accuracy, precision, recall, and F1 score between the zero-shot and few-shot learning with PEFT in NER (Fig. 3d, p > 0.05), there is a trend that few-shot learning performs above zero-shot learning.

### Results of Testing Hypothesis 5: Fine-tuning for One Clinical Domain May (or May Not) Impact Model Performance Across Other Clinical Domains in NER

To evaluate Hypothesis 5, we compared the performance of a clinical domain-specific PEFT model with zero-shot learning and its impact across other clinical domains in NER. For example, we conducted PEFT on agitation in dementia. We then compared the model’s impact on NER for malnutrition risk factors. We then compared the performance of a clinical domain-specific PEFT model with few-shot learning and its impact across other clinical domains, following the same pattern we did for zero-shot learning.

#### Comparing the Performance of a Clinical Domain-Specific PEFT Model with Zero-Shot Learning and Its Impact on Other Clinical Domains in NER

**Fig. 4:**
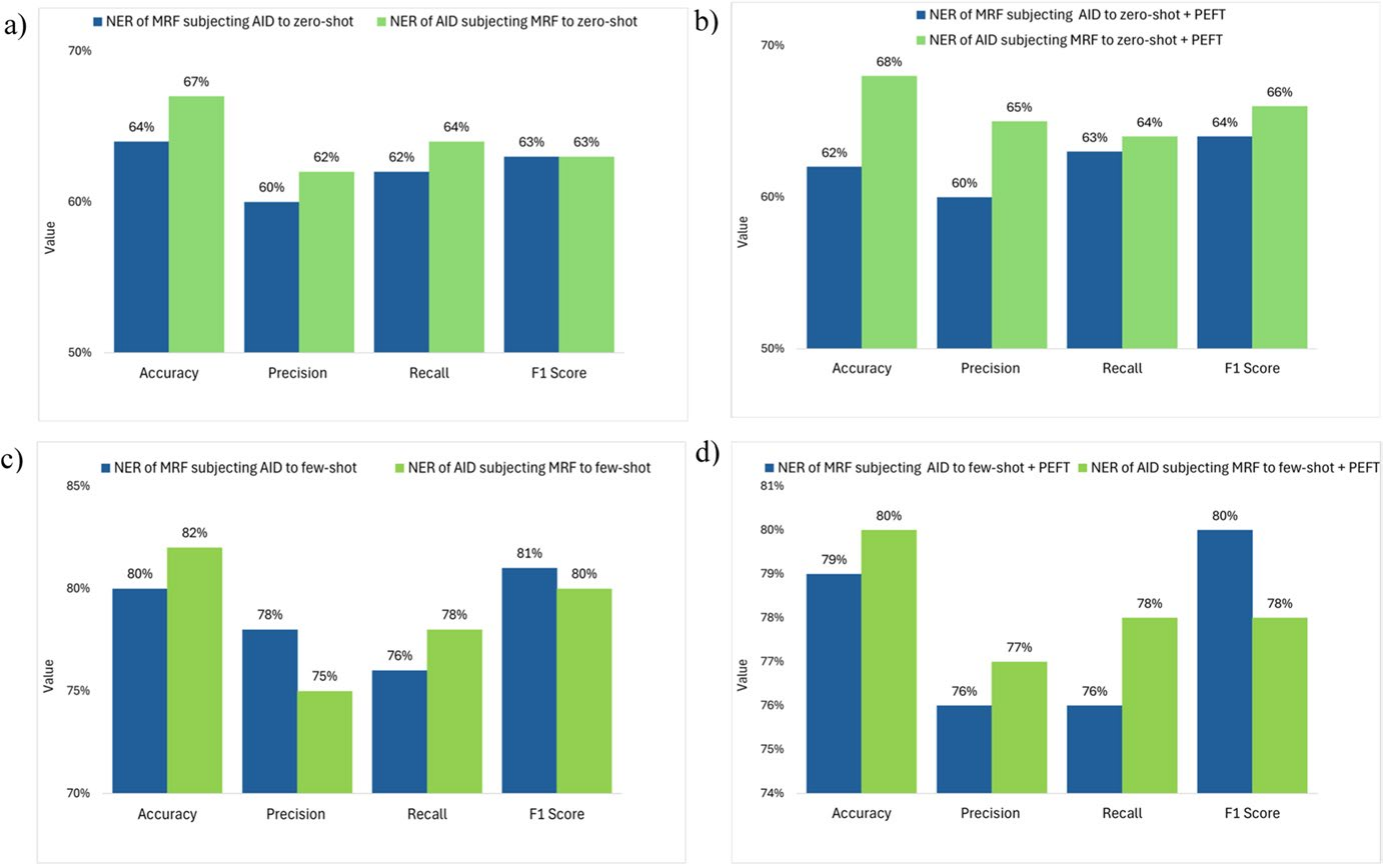
Performance of the measurement indicators for the other clinical domains in NER **a** when one clinical domain was only trained with zero-shot learning, **b** when training on one clinical domain with zero-shot learning and PEFT, **c** when one clinical domain was only trained with few-shot learning, and **d** when training on one clinical domain with few-shot learning and PEFT. Note: “IE” denotes with information extraction

No significant difference is found in accuracy, precision, recall, and F1 score for the other clinical domains between the two training models, pure zero-shot learning and zero-shot learning with PEFT for a specific clinical domain in NER. (see Fig. 4a, b, p > 0.05).

#### Comparing a Clinical Domain-Specific PEFT Model Performance with Few-Shot Learning and Its Impact on Other Clinical Domains in NER

No significant difference is found in accuracy, precision, recall, and F1 score for the other clinical domains between the two training models, pure few-shot learning and few-shot learning with PEFT for NER (see Fig. 4c, d, p > 0.05).

### Results of Testing Hypothesis 6: Retrieval Augmented Generation May (or May Not) Improve Both Zero-Shot and Few-Shot Learning Performance

To evaluate Hypothesis 6, we compared the performance of the model of (1) zero-shot learning without RAG and with RAG and (2) few-shot learning without RAG and with RAG for NER.

#### Comparing the Performance of the Zero-Shot Learning Model Without RAG and with RAG for NER

**Fig. 5:**
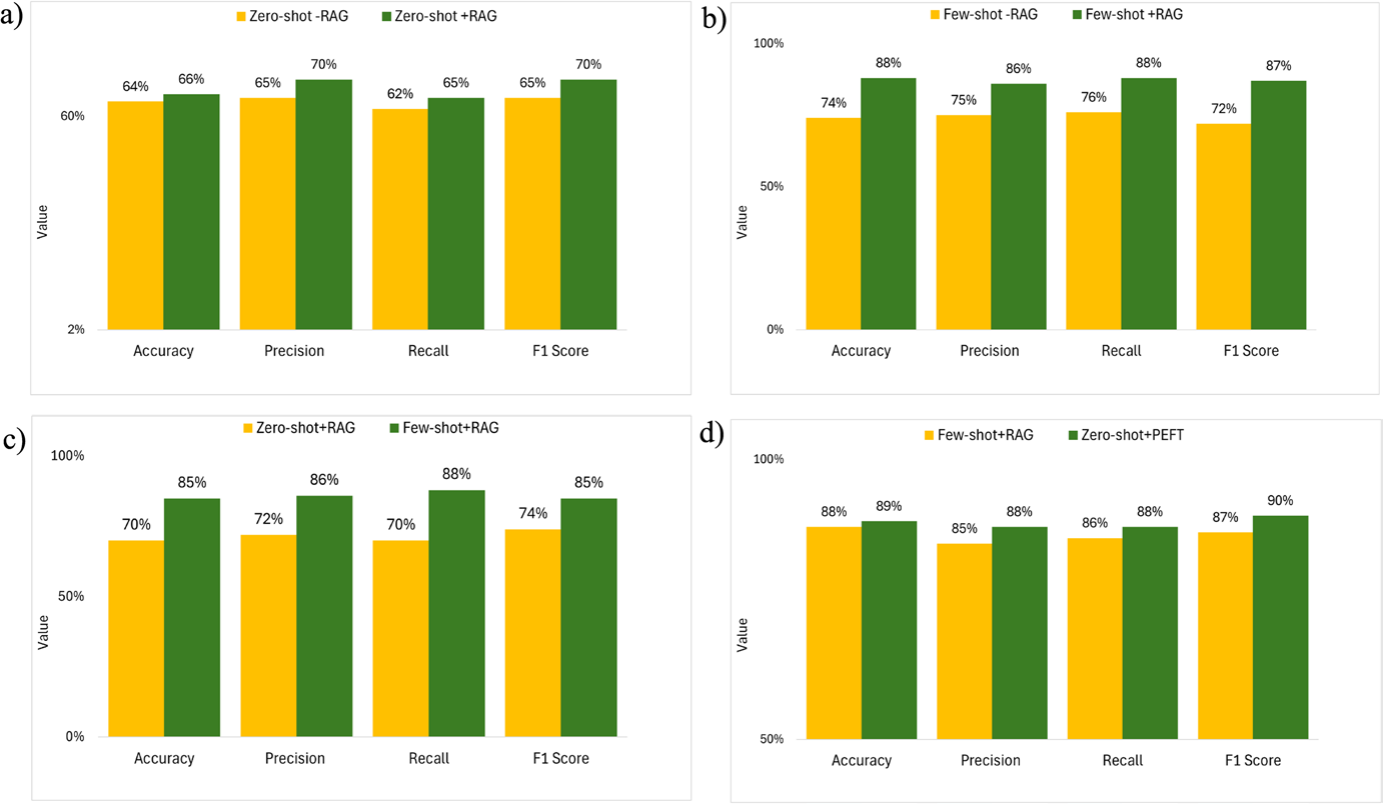
Comparative evaluation of model performance for NER with the following training methods: **a** zero-shot learning without RAG versus with RAG, **b** few-shot learning without RAG versus with RAG, **c** zero-shot learning with RAG versus few-shot learning with RAG, and **d** few-shot learning with RAG versus zero-shot learning with PEFT. Note: “-RAG” denotes without RAG

Although no statistically significant difference is found in accuracy, precision, recall, and F1 score between the zero-shot learning without RAG and with RAG in NER (Fig. 5a, p > 0.05), the results suggest a trend where zero-shot learning with RAG tends to perform better than without RAG.

#### Comparing the Performance of the Few-Shot Learning Model Without RAG and with RAG for NER

Few-shot learning with RAG significantly improves model accuracy, precision, recall, and F1 score in NER (Fig. 5b, p < 0.05). The level of improvement is as follows: a 19% increase in model accuracy, a 15% increase in precision, a 16% increase in recall, and a 21% increase in F1 score.

### Results of Testing Hypothesis 7: Zero-Shot Learning May (or May Not) Reach the Same Level of Performance as Few-Shot Learning for the NER with RAG

To evaluate Hypothesis 7, we compared the performance of zero-shot and few-shot learning with RAG for NER. Few-shot learning with RAG significantly improves model accuracy, precision, recall, and F1 score in NER than zero-shot learning (Fig. 5c, p < 0.05). The level of improvement is as follows: a 17% increase in model F1 score, an 18% increase in model accuracy, an 18% increase in precision, and a 23% increase in recall.

### Results of Testing Hypothesis 8: Few-Shot Learning with RAG May (or May Not) Perform Better than Zero-Shot Learning with PEFT for NER

To evaluate Hypothesis 8, we compared the performance of few-shot learning with RAG and zero-shot learning with PEFT for NER. No significant difference is found in accuracy, precision, recall, and F1 score between the few-shot learning with RAG and zero-shot learning with PEFT (Fig. 5d, p > 0.05) in NER. However, there is a trend that zero-shot learning with PEFT performs above few-shot learning with RAG.

## Discussion

This study explores the impact of zero-shot and few-shot prompt learning strategies, both with and without PEFT and RAG, on NER across two clinical domains. These include agitation in dementia and malnutrition risk factors. To achieve this, eight research hypotheses have been formulated, and experimental designs have been implemented to rigorously test these hypotheses.

Our first hypothesis proposed that zero-shot and few-shot learning with similar prompting templates exhibit comparable performance in NER across various clinical domains. Our findings support the alternative hypothesis, confirming that there is no significant difference in performance between these two learning approaches with similar prompting templates. This performance disparity may be attributed to the consistent design of prompting templates, which ensures that both approaches access and utilize relevant information in a similar manner.

Our second hypothesis proposed that few-shot learning performs better than zero-shot learning for the same NER. Our findings support the alternative hypothesis, confirming that few-shot learning significantly enhances performance compared to zero-shot learning (without PEFT or RAG). This improvement indicates that few-shot domain adaptation effectively minimizes false positives and false negatives while increasing true positives in NER tasks. These results highlight that exposing an LLM to initial information from the target domain can significantly improve its performance [58–60] in NER tasks. Our third hypothesis suggested that PEFT can improve both zero-shot and few-shot learning performance. The results support the alternative hypothesis, demonstrating that PEFT significantly improves performance in both learning modes compared to the models without PEFT. Our findings suggest that applying PEFT within a specific domain effectively reduces false positives and false negatives while increasing true positives in domain-specific NER tasks. This confirms that the PEFT approach facilitates targeted modifications to the model’s parameters [29, 30, 61], leading to improved overall performance in NER tasks within that domain.

Our fourth hypothesis proposed that zero-shot learning can reach the same level of performance as few-shot learning for the same NER after PEFT. Our findings support the alternative hypothesis, confirming no significant difference in performance between zero-shot and few-shot learning with PEFT. However, there is consistently a slight improvement with few-shot learning. This suggests that while additional examples in few-shot learning may not drastically enhance performance for domain-fine-tuned models, they still provide a marginal benefit, potentially refining the model’s contextual understanding. Our fifth hypothesis proposed that fine-tuning for one clinical domain impacts model performance across other clinical domains in NER. However, the findings support the null hypothesis, indicating that fine-tuning for a specific domain does not significantly affect the model’s performance on other domains. The rationale behind this lies in the methodology of PEFT, which focuses on training only a selective subset of the pre-trained model’s parameters [29, 61]. This insight suggests that the model can be effectively tailored to a specific clinical domain without compromising its effectiveness in handling diverse domains in NER. This adaptability underscores the potential of the PEFT approach within the LLM for various clinical domains.

Our sixth hypothesis asserts that RAG can improve both zero-shot and few-shot learning performance. However, the findings support the null hypothesis, showing no significant difference in performance between zero-shot learning with and without RAG. In contrast, a significant improvement was observed in few-shot learning when RAG was utilized. The lack of enhancement in zero-shot learning with RAG may be due to the absence of task-specific examples, which limits the model’s ability to leverage retrieved information and generalize to unseen tasks effectively [58, 59, 62]. Nevertheless, the results suggest that zero-shot learning with RAG consistently performs slightly better than without RAG. This could indicate that RAG offers a subtle advantage by providing additional context, though insufficient to result in substantial gains. Conversely, the significant performance boost in few-shot learning suggests that RAG effectively leverages the limited examples to enrich model understanding, improving classification accuracy. This disparity highlights RAG’s potential effectiveness in contexts where the model can benefit from supplementary information, particularly in few-shot settings [37, 63].

Our seventh hypothesis asserts that zero-shot learning can reach the same level of performance as few-shot learning for the same NER with RAG. Our findings support the null hypothesis, demonstrating a significant difference in performance between zero-shot and few-shot learning with RAG. Zero-shot learning may not reach the same performance level as few-shot learning with RAG due to the absence of task-specific examples that help the model better understand the nuances of the clinical tasks. It relies solely on the model’s pre-existing knowledge [58, 59], which may not cover the specific clinical details required for accuracy. In contrast, few-shot learning provides the model with critical initial information and context from the target domain, enabling it to make more informed predictions [37, 63, 64]. As a result, while RAG can enhance contextual relevance, the lack of direct examples in zero-shot learning limits its effectiveness compared to few-shot approaches.

Our eighth hypothesis proposed that few-shot learning with RAG and zero-shot learning with PEFT achieve the same performance levels in NER. The results support the alternative hypothesis, showing that both methods can deliver similar outcomes despite their distinct mechanisms. Zero-shot learning with PEFT selectively fine-tunes the most relevant parameters [29], enabling the model to adapt effectively to new domains with minimal labeled data [29, 61]. On the other hand, few-shot learning with RAG benefits from integrating relevant external information through retrieval, combined with task-specific examples [37, 64]. This enhances the model’s ability to handle complex clinical data, leading to fewer errors, such as false positives and false negatives—an insight derived from our study’s findings. Interestingly, the results show a slight improvement in performance with zero-shot learning using PEFT compared to few-shot learning with RAG. This marginal advantage may be attributed to the domain-specific fine-tuning inherent in PEFT, which enables the model to better leverage existing domain knowledge. Overall, our findings demonstrate that both PEFT and RAG excel in adapting to domain-specific NER tasks, highlighting their complementary strengths [65, 66] in improving model performance for IE in real-world clinical settings.

The findings of this study have significant implications for integrating AI models, particularly generative LLMs, into clinical practice. When combining both zero-shot and few-shot learning approaches with RAG and PEFT on IE tasks, the findings present efficient solutions for improving NER for clinical IE in various healthcare settings. Given the ongoing challenge of obtaining labeled data from domain experts, RAG, combined with few-shot learning, emerges as an efficient option. By leveraging external knowledge sources through retrieval, RAG minimizes dependence on large, annotated datasets, reducing the need for extensive fine-tuning specific to a task. This makes RAG a quicker and less resource-intensive solution, as it does not require the same level of GPU resources or training time as PEFT, which does not require high technical expertise [50]. In contrast, PEFT demands more computational power and expertise, requiring substantial GPU resources and additional time for fine-tuning specific model parameters [50]. However, the enhanced performance that PEFT offers makes it useful in scenarios where the computational infrastructure is available.

This study highlights the adaptability of RAG as an approach equally effective as PEFT in equipping generative LLMs to integrate domain-specific knowledge, reinforcing the pivotal role of NER in healthcare. The findings provide a solid foundation for deploying IE tools in clinical settings, guiding the choice of appropriate training methods, and ensuring their successful integration into RAC settings. The study contributes to several key areas: (1) geriatric care: NER models help identify residents at risk of malnutrition and other conditions like agitation in dementia, enabling early intervention and personalized care plans; (2) population health management: by extracting insights from clinical narratives, NER aids in analyzing health trends and improving healthcare strategies in RACFs; (3) clinical research and trial recruitment: NER ensures accurate retrieval of participant information from EHRs, streamlining the recruitment process; and (4) clinical decision support: NER enhances clinical decision-making by identifying critical information, such as symptoms and risk factors, leading to more accurate and efficient resident care.

This study encompasses four notable limitations. Firstly, our study encompasses two clinical domains for NER. However, we recognize that these tasks may not represent a diverse spectrum of clinical scenarios. In the future, we will broaden our scope by incorporating additional clinical domains for NER: depression in dementia and frailty index into our study. Secondly, although we have examined NER as the IE task, additional tasks, such as question answering, summarization, and relation extraction, are yet to be explored to enable a more comprehensive knowledge about the LLM’s performance in IE for free-text data. The third limitation relates to the selection of model performance evaluation metrics, which utilize accuracy, precision, recall, and F1 score as the primary evaluation metrics. In future studies, we will broaden our evaluation metrics to encompass calibration, robustness, fairness, bias, toxicity, and efficiency. Diversifying evaluation criteria will provide a more comprehensive and nuanced assessment of the model’s performance across various dimensions. This will enhance our findings’ reliability and applicability in real-world EHR applications.

## Conclusion

This study compares the performance of zero-shot and few-shot learning on NER tasks and the impact of PEFT and RAG on their performance. Our findings reveal that the same prompting template—whether zero-shot or few-shot, with or without PEFT or RAG—yields comparable performance across different clinical domains. Few-shot learning consistently outperforms zero-shot learning in NER tasks, even without PEFT or RAG. However, when PEFT is integrated with both zero-shot and few-shot learning, zero-shot learning achieves performance levels similar to few-shot learning in NER tasks. On the other hand, with RAG, few-shot learning consistently surpasses zero-shot learning, indicating RAG’s superior ability to provide relevant external knowledge in few-shot learning. Our analysis also shows that fine-tuning LLMs for a particular clinical domain does not significantly impact the model’s performance when applied to other clinical domains in NER. Notably, few-shot learning with RAG achieves performance comparable to zero-shot learning with PEFT, further highlighting the adaptability and efficiency of these advanced methods in clinical applications.

## Supplementary Information

Below is the link to the electronic supplementary material.

Supplementary file1 (DOCX 38 KB)

## Data Availability

No datasets were generated or analysed during the current study.
