# Supplementary material for: Adapting Generative Large Language Models for Information Extraction from Unstructured Electronic Health Records in Residential Aged Care: A Comparative Analysis of Training Approaches
Source: J Healthc Inform Res. 2025 Feb 20;9(2):191–219. doi: 10.1007/s41666-025-00190-z (PMC12037947; doi:10.1007/s41666-025-00190-z)
Supplement: Supplementary file 1 — Supplementary file1 (DOCX 38 KB) [file 41666_2025_190_MOESM1_ESM.docx]

**Adapting generative large language models for information extraction from unstructured electronic health records in residential aged care: a comparative analysis of training approaches.**

Dinithi Vithanage^1^, Chao Deng^2^, Lei Wang^1^, Mengyang Yin^3^, Mohammad Alkhalaf^1^, Zhenyu Zhang ^1^, Yunshu Zhu^1^, Ping Yu^1, *^

1. School of Computing and Information Technology, University of Wollongong, Wollongong, Australia
2. School of Medical, Indigenous and Health Sciences, University of Wollongong, Wollongong, Australia
3. Opal Healthcare, Sydney, Australia

ORCiD Id: Dinithi Vithanage <https://orcid.org/0000-0001-5851-7158>, Chao Deng <https://orcid.org/0000-0003-1147-5741>, Lei Wang <http://orcid.org/0000-0002-0961-0441>, Mengyange Yin <https://orcid.org/0000-0002-0212-4598>, Zhenyu Zhang <https://orcid.org/0000-0003-1853-4978>, Yunshu Zhu <https://orcid.org/0000-0003-2786-0775>, Ping Yu <https://orcid.org/0000-0002-7910-9396>.

**Correspondence:** ping@uow.edu.au

**Supplementary Table 1**

Example results generated from the final prompts.

| **Prompt** |
| --- |
| **###Task:** Review nursing progress notes for agitation symptoms in dementia  As a nursing expert, you are required to review a nursing progress note for a resident with dementia in a residential aged care facility. Your review should focus on identifying symptoms of agitation associated with dementia as documented in the note.  Symptoms of agitation in dementia:  Disruptive vocalisation, verbally aggressive behaviour, arguing, complaining, cursing, threat, using abusive language, using accusatory language, using foul language, using hostile language, using obscene language, using profane language, verbally nonaggressive behaviour, ceaseless talking, constant repetition of word, constant unwarranted requests for attention, constant unwarranted requests for help, constant unwarranted requests for reassurance, echolalia, groaning, grunting, howling, making bizarre noise, rambling, repetitive questioning, roaring, screaming, shouting, speaking in excessively loud voice, emotional distress, anger, frustration, irritability, mood swing, negativism, outburst, physically aggressive behaviour, biting, destroying property, fighting, grabbing, hitting, hurting self, hurting someone, kicking, pushing, resisting, scratching, shoving, slamming, spitting on people, staring, striking people, tearing, throwing object, physically nonaggressive behaviour, constant manipulation of object, fidgeting, gesturing, hand wringing, inappropriate dressing, inappropriate handling object, inappropriate undressing, pacing, pointing finger, repetitive physical mannerism, restlessness, rocking, rummaging, searching, wandering, bruxism, resisting, punching, absconding, calling out, physical agitation, facial grimacing, moving furniture, hoard items, intrusive of others privacy, gets up and down from constantly, urinating on the floor.  **###Instrcuctions:**   1. Identify symptoms: Examine the progress note to identify any symptoms of agitation, including those listed above. If symptoms that are not explicitly listed are observed, include them as well, ensuring they are relevant to agitation in dementia. 2. List identified symptoms: If agitation symptoms are evident, provide a complete and concise list of all identified symptoms.   Example cases:  Example 1:  Identified symptoms: physical agitation/aggression, verbal disruption, calling out, screaming  Progress note:  "Resident with vascular dementia exhibits confusion and disorientation alongside significant physical agitation/aggression and verbal behaviors, including verbal disruption, calling out, and screaming. These behaviors disturb others. Staff provide reassurance and distraction strategies such as music therapy and card games. During heightened distress, her daughter is contacted for support."  Example 2:  Identified symptoms: wandering, frightening others, arguing, refusing care  Progress note:  "Resident frequently wanders into others' rooms, mistaking them for her own. This behavior frightens others and results in arguments. The resident refuses care for hygiene and continence management, requiring staff intervention and redirection. Mealtimes involve guided supervision to ensure safety."  Example 3: Identified symptoms: punching  Progress note:  "Resident's advancing dementia has led to increased physical aggression, particularly punching during interactions with staff when she feels threatened or confused. Staff are implementing strategies to provide reassurance and maintain a safe environment."  Please use this structured approach to complete your review of the nursing progress note.  **###Input:** Resident experiences cognitive impairment due to dementia, resulting in a diminished ability to perform activities of daily living (ADLs) independently. Assistance is required for personal hygiene, toileting, and managing incontinence of both feces and urine. During mealtimes, the resident needs assistance and encouragement to eat, with occasional feeding as necessary. She adheres to a soft, bite-sized medium diet with thin fluids. The resident frequently engages in wandering behavior, necessitating gentle redirection. Additionally, she exhibits bruxism, often producing musical and repetitive sounds.  **###Response:** Identified symptoms:  wandering  bruxism  making musical, repeated sound |
| **###Task:** Review nursing progress notes for malnutrition risk factors in dementia  As a nursing expert, you are required to review a nursing progress note for a resident in a residential aged care facility. Your review should focus on identifying potential risk factors for malnutrition as documented in the note.  Potential malnutrition risk factors:  Anxiety, bowel blockage, cancer, chest infection, chronic wound, confusion, constipation, delirium, dementia, depression, diabetes, diarrhea, difficulty swallowing, dysphagia, eating disorder, food preference, frailty, gastritis, heart disease, HIV, hospital admission, isolation, kidney disease, liver disease, malabsorption medication, nausea, Parkinson, pneumonia, poor appetite, poor intake, poor oral health, pressure ulcer, sepsis, stroke, suboptimal intake, surgery, vomiting.  **###Instructions:**   1. Identify risk factors: Examine the progress note to identify any malnutrition risk factors, including those listed above. If risk factors not explicitly listed are observed, include them as well, ensuring they are relevant to malnutrition. 2. List identified risk factors: If any malnutrition risk factors are evident, provide a complete and concise list of the identified risk factors.   Example Cases:  Example 1:  Identified risk factor: confusion  Progress Note:  "Resident requires comprehensive assistance with his hygiene and toileting needs due to his confusion. He experiences incontinence of both urine and feces, necessitating the use of pads around the clock. A nurse is responsible for assisting him with toileting, changing his pads, cleansing his groin area, and applying barrier cream to mitigate the risk of skin issues or breakdown."  Example 2:  Identified risk factor: constipation  Progress note:  "Resident requires the assistance of a nurse for his hygiene and toileting needs, primarily due to his unsteady gait. He utilises pads due to incontinence issues. Additionally, he is prone to constipation and receives aperients as necessary. The current intervention measures in place have proven to be effective."  Please use this structured approach to complete your review of the nursing progress note.  **### Input:** Resident mobilizes with the assistance of a four-wheel walker (4WW) under staff supervision. Transfers are conducted with the supervision of one staff member to ensure safety, as the resident is considered a fall risk due to her tendency to rush. Personal hygiene is assisted by one staff member due to confusion.  **### Response:** Identified risk factor: confusion |

**Supplementary Table 2**

P values for comparative analysis of model outputs for two clinical tasks - agitation in dementia and malnutrition – using various training methods.

| Method | Accuracy | Precision | Recall | F1 score |
| --- | --- | --- | --- | --- |
| Zero-shot learning | 0.36 | 0.31 | 0.34 | 0.33 |
| Zero-shot learning with PEFT | 0.08 | 0.09 | 0.08 | 0.075 |
| Zero-shot learning with RAG | 0.38 | 0.29 | 0.30 | 0.28 |
| Few-shot learning | 0.21 | 0.36 | 0.30 | 0.28 |
| Few-shot learning with PEFT | 0.6 | 0.30 | 0.28 | 0.33 |
| Few-shot learning with RAG | 0.38 | 0.32 | 0.36 | 0.39 |
| Comparing zero-shot learning versus few-shot learning | 3.2-7e | 2.8-5e | 2.0-7e | 1.5-7e |
| Comparing zero-shot learning versus zero-shot learning with PEFT | 3.35-5e | 3.35-5e | 2.12-5e | 2.0-5e |
| Comparing few-shot learning versus few-shot learning with PEFT | 2.35-5e | 2.35-5e | 1.82-5e | 2.10-5e |
| Comparing zero-shot learning with PEFT versus few-shot learning with PEFT | 2.32 | 2.32 | 1.85 | 1.84 |
| Comparing zero-shot learning versus zero-shot learning with RAG | 0.46 | 0.42 | 0.48 | 0.40 |
| Comparing few-shot learning versus few-shot learning with RAG | 2.00-3e | 1.79-4e | 2.25-3e | 2.13-3e |
| Comparing zero-shot learning with RAG versus few-shot learning with RAG | 2.24-3e | 2.24-3e | 1.02-5e | 1.45-2e |
| Comparing few-shot learning with RAG versus zero-shot learning with PEFT | 1.22 | 1.22 | 0.89 | 1.14 |

**Supplementary Table3**

Three training rounds' mean value and standard deviations for one machine learning method.

| Method | Accuracy | Precision | Recall | F1 score |
| --- | --- | --- | --- | --- |
| Zero-shot learning | 63.51% (28.21%) | 64.55% (26.41%) | 62.21% (29.29%) | 64.70% (27.84%) |
| Few-shot learning | 73.68% (22.12%) | 74.61% (18.45%) | 76.01% (20.42%) | 72.42% (19.22%) |
| Zero-shot learning with PEFT | 89.81% (11.82%) | 88.22% (14.81%) | 88.53% (13.36%) | 86.44% (12.81%) |
| Few-shot learning with PEFT | 90.82% (11.52%) | 90.41% (12.10%) | 87.92% (12.11%) | 89.74% (13.41%) |
| Zero-shot learning with RAG | 66.22% (24.22%) | 70.49% (19.17%) | 65.22% (26.22%) | 70.11% (19.14%) |
| Few-shot learning with RAG | 88.21% (13.88%) | 86.48% (14.22%) | 88.47% (13.82%) | 87.25% (12.01%) |
